# Upper gastrointestinal dysmotility after spinal cord injury: is diminished vagal sensory processing one culprit?

**DOI:** 10.3389/fphys.2012.00277

**Published:** 2012-07-17

**Authors:** Gregory M. Holmes

**Affiliations:** Neural and Behavioral Sciences, Penn State University College of MedicineHershey, PA, USA

**Keywords:** brain-gut axis, gastric emptying, gastric motility, thoracic spinal cord injury, vago-vagal reflexes

## Abstract

Despite the widely recognized prevalence of gastric, colonic, and anorectal dysfunction after spinal cord injury (SCI), significant knowledge gaps persist regarding the mechanisms leading to post-SCI gastrointestinal (GI) impairments. Briefly, the regulation of GI function is governed by a mix of parasympathetic, sympathetic, and enteric neurocircuitry. Unlike the intestines, the stomach is dominated by parasympathetic (vagal) control whereby gastric sensory information is transmitted via the afferent vagus nerve to neurons of the nucleus tractus solitarius (NTS). The NTS integrates this sensory information with signals from throughout the central nervous system. Glutamatergic and GABAergic NTS neurons project to other nuclei, including the preganglionic parasympathetic neurons of the dorsal motor nucleus of the vagus (DMV). Finally, axons from the DMV project to gastric myenteric neurons, again, through the efferent vagus nerve. SCI interrupts descending input to the lumbosacral spinal cord neurons that modulate colonic motility and evacuation reflexes. In contrast, vagal neurocircuitry remains anatomically intact after injury. This review presents evidence that unlike the post-SCI loss of supraspinal control which leads to colonic and anorectal dysfunction, gastric dysmotility occurs as an indirect or secondary pathology following SCI. Specifically, emerging data points toward diminished sensitivity of vagal afferents to GI neuroactive peptides, neurotransmitters and, possibly, macronutrients. The neurophysiological properties of rat vagal afferent neurons are highly plastic and can be altered by injury or energy balance. A reduction of vagal afferent signaling to NTS neurons may ultimately bias NTS output toward unregulated GABAergic transmission onto gastric-projecting DMV neurons. The resulting gastroinhibitory signal may be one mechanism leading to upper GI dysmotility following SCI.

## Introduction

The association of gastrointestinal (GI) pathology with neurological trauma dates back to the mid-nineteenth century observations of Rokitansky and subsequent descriptions by Schiff [both cited by Cushing (1932)]. Gastric stasis and ulceration, commonly known as “Cushing's ulcer,” associated with severe trauma has been repeatedly described in the clinical literature in the 80 years since Harvey Cushing's original report. Regardless of the nature of the original traumatic insult, the absence of abnormal GI motility is a strong predictor of patient outcome and length of hospitalization.

Roughly 11,000 new spinal cord injury (SCI) cases occur each year and the current United States SCI population has been estimated as high as 1.2 million people (*The Christopher and Dana Reeve Paralysis Resource Center*). In addition to the immediate loss of sensation and motor function, SCI also profoundly affects the autonomic nervous system (Weaver et al., 2006; Inskip et al., 2009; Krassioukov, 2009). While attention to autonomic dysfunction has increased in recent years, studies targeting cardiovascular and bladder dysfunction outnumber those of GI dysfunction. While the former derangements present formidable challenges to the SCI individual, GI complications are typically responsible for 11% of hospitalizations in the SCI population (Middleton et al., 2004; Jaglal et al., 2009) and are consistently rated as serious quality of life issues (Anderson, 2004).

Functional GI motility disorders present as a broad range of symptoms which include delayed gastric emptying, early satiety and the sensation of nausea, bloating, abdominal pain and diminished propulsive transit along the entire length of the GI tract. Due to the segmental distribution of the spinal neurocircuitry regulating both visceral preganglionic and somatic motor neurons, the degree of disability, morbidity, and mortality following injury tends to be associated with the spinal level at which injury occurred.

Conventional division of the GI tract into designations of upper and lower compartments remains open to debate. Based upon embryological development, the GI tract can be classified along three divisions consisting of (1) the foregut, which gives rise to the esophagus, stomach, and duodenum as far as the major duodenal papilla; (2) the midgut, from where the bile duct enters at the major duodenal papilla to the mid-transverse colon; and (3) the hindgut, from the mid-transverse colon to the anus. For the present purposes of this review, the upper GI tract is defined as the esophagus, stomach and proximal duodenum. The scope of this review precludes discussion of post-SCI pathologies along the entire GI tract. This is especially true for the intestines and rectoanal junction, since the loss of descending input to the sympathetic, parasympathetic, and pudendal nuclei located throughout the thoracic, lumbar, and sacral spinal cord presents a pattern of neural control that is fundamentally different from upper GI innervation.

## Neural control of upper gastrointestinal function

The principal nutritive functions of the GI tract, the digestion, absorption and propulsion of nutrients, and the maintenance of proper fluid balance, are critically dependent upon a hierarchy of enteric, parasympathetic and sympathetic neural control.

The enteric nervous system (ENS) provides powerful control over the smooth musculature, secretory glands and microvasculature of the digestive tract [reviewed in Woods (2004)]. This so-called “mini-brain” of the gut is comprised of primary afferent neurons, interneurons, and efferent neurons that are capable of complete reflex activities and quasi-autonomous control of digestion. The ENS mediates digestion through localized control over the individual reflex systems and by integrating the actions of these effectors along the GI tract into organized patterns of digestion. Without this localized autonomous control, proper digestive processes do not occur (De Giorgio and Camilleri, 2004). However, while the intrinsic reflexes necessary for proper intestinal function are mediated by the ENS, these digestive processes must ultimately integrate with the homeostatic needs of the entire organism through brain-gut connections.

**Figure 1:**
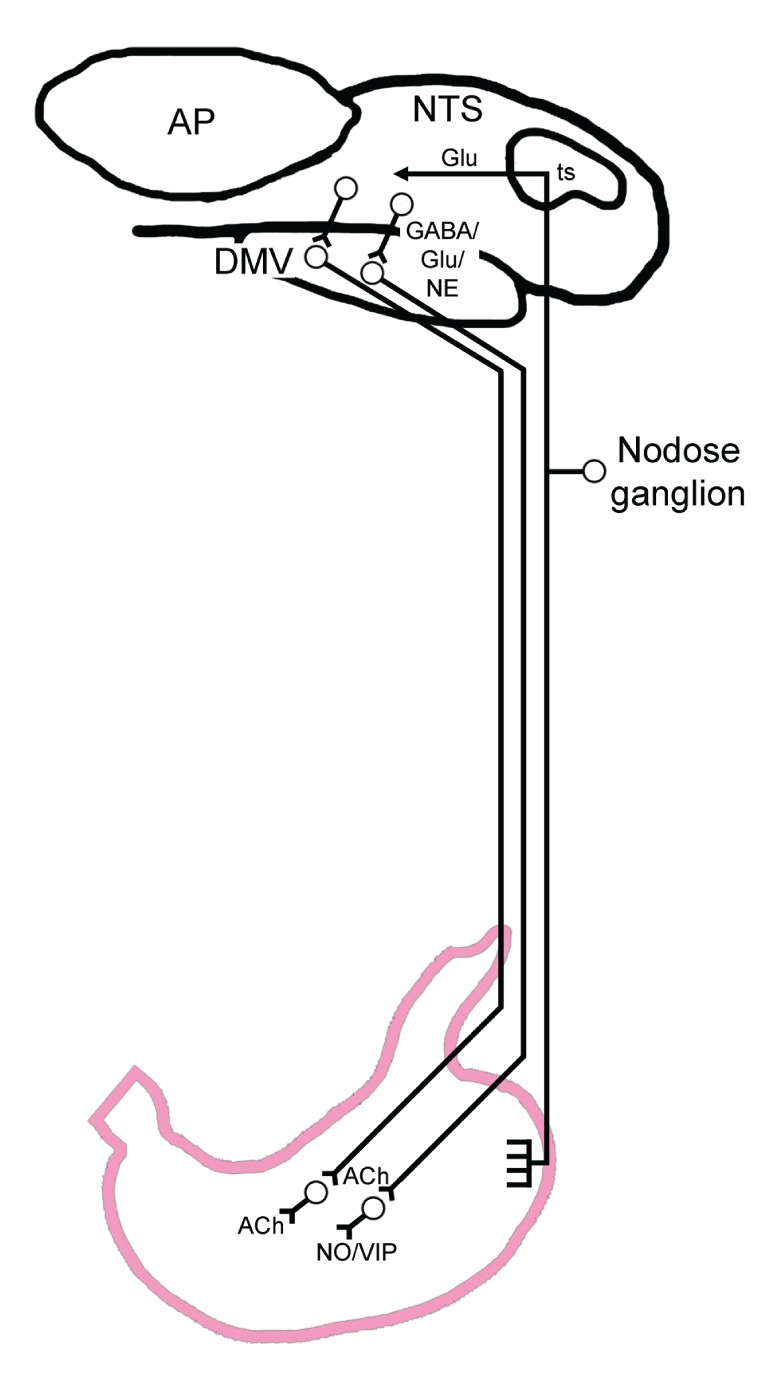
**General visceral afferent signals are transmitted to the brainstem by afferent fibers distributed throughout the proximal GI tract that traverse within the vagus nerve.** The *dashed line* arbitrarily indicates the transition between the fundus (orally) and the corpus (caudally). The cell bodies for these vagal afferents reside within the nodose ganglion. Vagal afferents enter the brainstem by way of the tractus solitarius (ts) and terminate principally as a glutamatergic (Glu) synapse onto second order neurons within the nucleus tractus solitarius (NTS). At the level of the NTS, converging projections from higher CNS centers (not pictured) are integrated and relayed by NTS neurons to regions which include the parasympathetic preganglionic neurons of the dorsal motor nucleus of the vagus (DMV) using the neurotransmitters GABA, glutamate or norepinephrine (NE). Together with the area postrema (AP) the NTS and DMV form the region of the dorsal vagal complex. Preganglionic DMV motor neurons innervate gastric enteric neurons by way of two competing pathways. Activation of one pathway initiates the cholinergic (Ach) mediated excitation of gastric smooth muscle which is necessary for gastric tone and motility. Alternatively, activation of a non-adrenergic, non-cholinergic (NANC) pathway exerts a profound gastric relaxation through the release of nitric oxide (NO) or vasoactive intestinal polypeptide (VIP). Reduction in gastric tone and motility, therefore, can be produced by either the withdrawal of excitatory cholinergic drive, or activation of NANC-mediated inhibition.

Unlike the small and large intestines, the ENS of the stomach lacks the capacity to independently control the moment-to-moment changes necessary for appropriate ingestive, milling, and emptying reflexes. While vago-vagal reflex circuits modulate digestive processes from the oral cavity to the transverse colon, the level of vagal control diminishes caudally. Gastric function is dominated by extrinsic neural circuits residing within the brainstem that modulate the gastric ENS neurocircuitry. Specifically, the extrinsic brainstem integration of gastric reflex function is centered within circuits of the dorsal vagal complex (DVC), which comprises the area postrema (AP), the nucleus tractus solitarius (NTS) and the dorsal motor nucleus of the vagus (DMV; Figure 1) (Travagli et al., 2006). The integrative activity that occurs within the DVC nuclei is the result of inputs originating from higher central nervous system (CNS) areas (Blevins et al., 2004; Morton et al., 2005; Blevins and Baskin, 2010); from spinosolitary inputs (Menetrey and Basbaum, 1987; Menetrey and de Pommery, 1991; Gamboa-Esteves et al., 2001) as well as from neurohormonal signals from the periphery. This latter-most signaling pathway occurs as a function of the fenestrated capillaries within the DVC that permit diffusion of circulating neuromodulators across a “leaky” blood brain barrier (Gross et al., 1990). All of these signals finely tune the coordinated emptying of nutrients from the stomach.

### Vagal afferent signaling

Details regarding the sensory innervation of the gut have been reviewed previously (Beyak et al., 2006). Briefly, the cell bodies of vagal afferent (sensory) fibers, including those that innervate the proximal GI tract, are located within the nodose ganglion (Browning and Mendelowitz, 2003). Afferent information originating in the gut terminates directly upon second order NTS neurons by way of a glutamatergic synapse (Hornby, 2001). These GI afferents can be categorized based upon two essential receptor qualities. Mechanosensitive receptors in the form of intraganglionic laminar endings (IGLEs; Powley and Phillips, 2002) and, possibly, intramuscular arrays (IMAs) (Berthoud and Powley, 1992) innervate the muscle layers in a manner consistent for the transduction of contractile and shearing forces (Powley and Phillips, 2002). Vagal IGLE innervation is densest in the esophagus and proximal-most portions of the GI tract (Berthoud et al., 1997; Neuhuber et al., 1998; Wang and Powley, 2000) and viseral sensory afferents terminate topographically within the subnuclei of the NTS (Altschuler et al., 1992). Specifically, esophageal sensory receptors that include IGLE's project exclusively to the subnucleus centralis (NTSc) (Cunningham and Sawchenko, 1990; Sengupta, 2000). As will be seen later, these exclusive esophageal projections to the NTSc provide a very unique model of a pure vago-vagal gastric reflex.

The second principal receptor classes are chemosensitive vagal afferents, particularly those within the lamina propria and distributed throughout the villi, which have been described throughout the GI mucosa (Berthoud et al., 1995). The specific response characteristics, ligands, and signal transduction pathways utilized by these vagal afferents are too extensive to be reviewed herein. However, two particular examples of luminal signaling have received considerable attention. One of the best characterized peptides controlling gastric and digestive functions is cholecystokinin (CCK), which is released from so-called I-cells within the proximal small intestine in response to fat or protein content of a meal (Dockray, 2006). CCK release has profound inhibitory effects on GI functions (Ritter et al., 1994; Moran and Kinzig, 2004; Woods, 2004), and its vagal mechanisms of action commonly ascribed to include a paracrine activation of vagal afferent fibers along the gut wall [reviewed in Raybould (2007)].

The GI neurohormone ghrelin, secreted from oxyntic cells within the gastric mucosa (Date et al., 2000; Grönberg et al., 2008), is up-regulated during periods of negative energy balance, such as before meals, and is down-regulated after feeding (Cummings et al., 2001). In animals and humans, ghrelin and ghrelin agonists exert profound stimulatory effects upon gastric motility and acid secretion as well as food intake and energy metabolism (Masuda et al., 2000; Levin et al., 2006; Tack et al., 2006; Ariga et al., 2007, 2008; Wang et al., 2008; Ejskjaer et al., 2009; Kobashi et al., 2009) though an inhibitory effect has been reported for fundic tone (Kobashi et al., 2009). Ghrelin has received considerable clinical interest as an endogenous stimulant of gastric motility (Nass et al., 2011; Stengel and Taché, 2012). Peripherally, ghrelin is considered to exert its gastroexcitatory effect on a vagally-mediated pathway which involves growth hormone secretagogue receptors that originate in the nodose ganglion and are transported to vagal afferent terminals (Date et al., 2002). The role of afferent fibers of the gastric vagal circuit was confirmed physiologically in that peripherally administered (i.e., circulating) ghrelin diminishes vagal afferent activity while vagotomy, midbrain transection, or perivagal capsaicin abolishes ghrelin-mediated facilitation of feeding, GH secretion, as well as activation of neuropeptide Y (NPY)- and growth hormone-releasing hormone (GHRH)-producing neurons (Date et al., 2002, 2006).

Many of the effects of endogenous gut peptides are mediated via a paracrine activation of the peripheral endings of vagal afferent fibers as described above. However, based upon a large body of work with CCK, mounting evidence has shown that GI peptides exert physiologically relevant actions when applied to central GI neurocircuitry (Talman et al., 1991; Branchereau et al., 1992, 1993; Blevins et al., 2000, 2004; Lin et al., 2004; Appleyard et al., 2005; Baptista et al., 2005b, 2006; Wan et al., 2007; Kobashi et al., 2009) including direct actions upon nodose ganglion (Blackshaw and Grundy, 1990; Simasko and Ritter, 2003), and DVC neurons (Baptista et al., 2005b; Zheng et al., 2005; Holmes et al., 2009a,b). Similarly, ghrelin receptor expression has been reported within the medullary brainstem and brainstem application of ghrelin exerts behavioral (Faulconbridge et al., 2003), gastric (Kobashi et al., 2009) and cardiovascular (Lin et al., 2004) responses. These studies suggest that functional CCK and ghrelin receptors are present on the vagal afferent terminals, the neuronal membrane and nerve terminals of subgroups of the NTS as well as on gastric-projecting DMV neurons. The source of feeding-related peptides acting centrally upon DVC neurocircuitry may be through local neuronal release or through the circulation, since the DVC has a leaky blood-brain barrier (Gross et al., 1990; Cottrell and Ferguson, 2004). The exact mechanism of gut peptides activating these neural circuits within the DVC remains to be elucidated. However, the GI effects of peptides such as CCK-8s (the sulfhated, octapeptide variant of CCK) may not be limited to a paracrine activation of the peripheral terminal of vagal afferent fibers but through direct effects on brainstem circuits which must be considered in physiological studies of neurally intact as well as injured preparations.

### Vagal efferent signaling

With regard to the stomach, motor output originating in the DMV regulates a complex interplay between two separate postganglionic circuits. While a small percentage of visceral afferent inputs modulate gastric reflex function by directly synapsing onto DMV neurons (Renehan et al., 1995), the majority of afferent signaling is directed to second order neurons within the NTS through a glutamatergic synapse (see Browning and Travagli, 2010). In turn, three distinct neurochemical phenotypes (glutamatergic, GABAergic and noradrenergic) of NTS neurons synapse onto DMV neurons. Emerging evidence following application of GABAergic antagonists to the DVC, strongly suggests that NTS GABAergic inputs onto DMV neurons tonically regulate the basal motor outflow to the stomach (Sivarao et al., 1998; Browning and Travagli, 2001; Herman et al., 2009, 2010; Babic et al., 2011), whereas glutamatergic and noradrenergic antagonism has little effect (Saltzstein et al., 1995; Soret et al., 2010).

As with all parasympathetic preganglionic neurons, DMV neurons are cholinergic and activate postganglionic neurons via actions at a nicotinic receptor. Gastric projecting neurons within the DMV exhibit a basal rate of spontaneous action potentials (1–2 Hz); (Travagli et al., 1991; Marks et al., 1993; Browning et al., 1999) which is modulated, though not generated, by synaptic inputs beyond those described for the NTS. Modulation of this spontaneous, low frequency DMV firing regulates an excitatory (cholinergic) circuit that is ultimately important to the antral milling of ingested solids and the delivery of reduced particles to the duodenum (Malagelada and Azpiroz, 1989). Gastric relaxation can occur as a consequence of inhibiting this tonically firing excitatory pathway (Abrahamsson and Jansson, 1969; Abrahamsson, 1973; Gillis et al., 1989; McCann and Rogers, 1992, 1994). However, activation of vagal afferents produces a potent gastroinhibition by also activating a non-adrenergic non-cholinergic (NANC) inhibitory vagal projection to the stomach mainly by the release of nitric oxide (NO) (Jansson, 1969; Abrahamsson, 1973; Takahashi and Owyang, 1997; Krowicki et al., 1999) though purinergic, and vasoactive intestinal polypeptide mechanisms have also been identified [reviewed in Chang et al. (2003)].

## Upper GI dysfunction after human SCI

### Esophageal function

The principal functions of the esophagus are centered on (1) the propulsion of ingesta to the stomach; and (2) prevention of gastroesophageal reflux of stomach contents. Esophageal motor control is a complex interplay of proximal striated musculature which is a combination of voluntary and reflexive control proximally that progressively yields to involuntary smooth muscle contractions distally.

Despite the clinical implications regarding the risk of aspiration that accompanies esophagogastric reflux, such that intensive management of the airway is also required (Kirshblum et al., 2002), there are relatively few reports addressing esophageal function following SCI. Based upon barium contrast imaging, early reports noted that the incidence of gastroesophageal reflux and hiatal hernia were limited to persons with SCI greater than 5 years duration (Gore et al., 1981). Later evidence, based upon subject questionnaire and endoscopic follow-up, reported a higher incidence of heartburn and esophageal chest pain in SCI subjects which was accompanied by endoscopic and histological evidence of esophagitis as well as diminished esophageal contractility (Stinneford et al., 1993). Diagnoses of dysphagia in cervical SCI population confirmed these earlier reports, but also identified the potential causal relationship between dysphagia and both artificial ventilation techniques (including tracheotomy) as well as anterior versus posterior approaches during spinal stabilization (Wolf and Meiners, 2003). Finally, to test the role of diaphragmatic crura upon gastroesophageal reflux containment, comparisons have been made between quadriplegic and paraplegic subjects. While cervical injury did significantly increase subjective reflux ratings, high-level injury did not predispose subjects to differences in endoscopic, manometric, or histological indications of esophagogastric abnormalities (Silva et al., 2008). Thus, the prevalence and potential mechanism of esophageal dysfunction after human SCI remains largely unresolved.

### Gastric function

The principal functions of the stomach are centered on (1) a reservoir component for ingested solids and liquids; (2) reduction of the size of food particles through both digestive secretions and the mechanical milling evoked by gastric contraction and relaxation; and (3) the feed-back mediated propulsion of ingesta into the duodenum. The gastric compartment can be subdivided into the fundus, which serves as reservoir and regulates intragastric pressure, and the more muscular corpus where food is churned until reduced in size in order for contraction of the antrum to facilitate passage through the pylorus leading to the duodenum.

Peptic ulceration has been reported following SCI (Tanaka et al., 1979) and in other traumatic injuries requiring intensive care. The prophylactic administration of proton pump inhibitors or histamine-2 receptor antagonists is widely employed in the ICU and may minimize this particular co-morbidity, though debate remains whether such practices are justified (Jung and MacLaren, 2002; Stollman and Metz, 2005). Less well characterized, and managed, are alterations in the motor components of gastric reflex function. Despite utilizing a variety of technical approaches, the majority of reports in the clinical literature describe derangements in upper GI reflex emptying and motility, especially after SCI occurring above the mid-thoracic spinal cord (Kewalramani, 1979; Berlly and Wilmot, 1984; Fealey et al., 1984; Nino-Murcia and Friedland, 1991; Rajendran et al., 1992; Stinneford et al., 1993; Segal et al., 1995; Kao et al., 1999; Williams et al., 2012). In extreme cases, the high degree of gastric feeding intolerance demonstrated by these patients necessitates aggressive total parenteral nutrition and occasionally invasive GI surgical intervention in order to maintain positive energy and nitrogen balance (Dwyer et al., 2002).

The GI neurohormone ghrelin, previously described as being secreted from oxyntic cells within the gastric mucosa (Date et al., 2000; Grönberg et al., 2008), is up-regulated during periods of negative energy balance, such as before meals, and is down-regulated after feeding (Cummings et al., 2001). Recently, a study of uninjured, paraplegic and quadriplegic individuals reported no differences in levels of serum ghrelin across all groups following an overnight fast (Wang et al., 2005). Unfortunately, with the exception of a study on eating attitudes (Levin et al., 2006), clinical studies are lacking regarding the dietary behaviors of the SCI population. In contrast to the able-bodied population, data regarding the orexigenic and pro-motility responses to exogenous ghrelin following SCI is non-existent.

### Proximal duodenal function

The principal functions of the proximal duodenum include (1) neutralization of acid in the chyme delivered from the stomach; and (2) enzymatic reduction of particles to simple molecules for absorption. Considerable feedback mechanisms exist between the antrum, pylorus, and duodenum for the delivery of chyme in a manner that does not exceed the digestive capacity of the small intestine (Ueno et al., 2005; Schulze, 2006). As a result of normal exposure to both an appropriate composition of reduced food particles and the rate of trans-pyloric delivery of chyme, the duodenum releases GI peptides and hormones that are integral to these feedback mechanisms (Dockray, 2006; Englander and Greeley, 2008; Dockray and Burdyga, 2011).

Data regarding gut hormone levels in the SCI population are scarce. Motilin, a 22-amino acid peptide released from the upper intestine, stimulates gastric and intestinal myoelectric activity during phase III contractions of the migrating myoelectric complex of the interdigestive phase. Comparisons of serum motilin levels in a limited sample of uninjured, paraplegic and quadriplegic subjects revealed that motilin levels were largely similar across all three groups, though there was a trend toward elevated motilin levels in the paraplegic group (Saltzstein et al., 1995). Peptide YY (PYY) is a 36-amino acid peptide hormone that is similar to GI peptides PP and NPY that is released from epithelial cells within the ileum and colon. However, the actions of circulating PYY target the upper GI as an “ileal brake” whereby PYY potently diminishes gastric acid secretion, gastric emptying, intestinal propulsion and pancreatic exocrine secretion (Englander and Greeley, 2008). In this same study described above, levels of PYY in chronic SCI individuals were similar in the fasted state but were significantly elevated in the early postprandial state of quadriplegic subjects (Saltzstein et al., 1995). The limited sample size and the absence of essential physiological parameters, such as gastric emptying rates during the postprandial serum measurements, limit the interpretation of these findings and merit re-evaluation in a larger sample from the SCI-population.

## Upper gastrointestinal dysfunction in experimental models of SCI

Over the past 20 years, the SCI research community has gained a clearer understanding of the interrelated cellular and biochemical processes which comprise the aftermath of SCI; identified the challenges for successful regeneration of damaged tissue; and expended considerable intellectual capital upon the recovery of stepping and standing after SCI. Unfortunately, post-SCI changes to GI autonomic reflexes remain inadequately explored, however, recent attention has been directed at derangements in GI function in animal models of experimental SCI.

Published reports and our own preliminary observations in an animal model of SCI have demonstrated striking similarities to the clinical presentation of profound GI dysmotility in humans. Expanding upon the initial reports that high thoracic spinal transection delayed the emptying of a phenol red liquid test meal (Gondim et al., 1999, 2001), we reported that T3-SCI animals show a diminished food intake (Primeaux et al., 2007). Subsequent studies utilized an Inactin-anesthetized preparation in which sub-miniature, dual-element, strain gauges were sutured to the serosal surface of the gastric corpus, thus recording circular smooth muscle contractions. We reported a significant reduction in gastric motility that was not altered by sympathectomy (Tong and Holmes, 2009). Our results were in agreement with earlier conclusions by Gondim et al. who concluded that post-SCI dysmotility was vagally mediated (Gondim et al., 2001). Furthermore, utilizing the [^13^C]-octanoate breath test see Ghoos et al. (1993) as an indirect measure of gastric emptying in intact rats, we concluded that diminished gastric function after SCI was not likely due to the effects of Inactin anesthesia (Qualls-Creekmore et al., 2010b).

**Figure 2:**
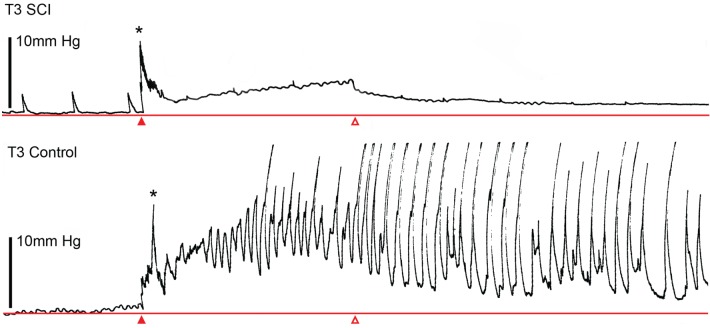
**Spinal cord injury diminishes mechanical sensitivity of the stomach to fluid distension.** Representative gastric pressure traces in high thoracic spinal cord injured (T3 SCI, *upper trace*) and surgical (laminectomy only) controls (T3 Control, *lower trace*) demonstrating that during 6 min of continuous filling (at a rate of 1 ml/min, starting at closed arrowhead and terminating at open arrowhead). T3 SCI rats exhibited a smaller increase in gastric pressure and that pressure-evoked motility waves were less pronounced. Initial pressure peak (denoted by asterisks) was an artifact of initiating the filling cycle. Gastric distension was maintained at the termination of the filling cycle. Distension was performed by passing a saline-filled catheter via an incision in the proximal duodenum and through the occluded pylorus. The lower esophageal sphincter was untouched and maintained closure at the gastric cardia.

Canon and Lieb were the first to describe the gastric accommodation reflex (Canon and Lieb, 1911). This vagally-mediated reflex is elicited either by physiological (low pressure) distension of the esophagus (Saltzstein et al., 1995)or direct filling of the isolated stomach (Takahashi and Owyang, 1997)and permits the stomach to relax in response to large volumes of ingesta, thus maintaining low levels of intragastric pressure (Wilbur and Kelly, 1973). In our T3-SCI model, physiological distension of the esophagus failed to elicit a reflex relaxation of the stomach (Tong and Holmes, 2009). Expanding upon the study by Takahashi and Owyang (Takahashi and Owyang, 1997), retrograde gastric filling in T3 surgical control rats elicited marked increases in intragastric pressure as well as intragastric pressure waves similar to those observed in conscious, freely moving rats fed a test meal (Janssen et al., 2008). In T3-SCI rats, both intragastric pressure, as well as intragastric pressure waves, are substantially diminished (Figure 2; Holmes et al., 2008).

Further studies using [^13^C]-octanoate tagged solid meals in awake animals confirmed that gastric dysmotility is accompanied by a delay in gastric emptying and that dysmotility persists up to 6 weeks after T3-SCI, (Qualls-Creekmore et al., 2010a). These persistent deficits led us to conclude that delayed gastric emptying is unlikely to be due to “spinal shock” as gastric dysmotility persists long after SCI animals are generally considered to have stabilized (ca. 3–6 weeks post-SCI).

Finally, with the understanding that the reflex control of the stomach is under considerable modulation by gut hormones, like CCK and ghrelin, we have begun to test the sensitivity of T3-SCI rats to GI peptides which evoke vagally-mediated gastric reflexes (Ueno et al., 2005). It is well accepted that peripheral CCK-8s activates C-type vagal afferent fibers and increases *c-Fos* immunoreactivity in NTS cells of neurally intact animals (Renehan et al., 1995; Zittel et al., 1999; Sullivan et al., 2007). Our study was particularly revealing in that peripheral CCK-8s administration 3 days after injury induced significantly less *c-Fos* expression in the NTS than in uninjured control rats (Tong et al., 2011). In the same experimental animals, *c-Fos* expression within the adjacent AP was similar in both groups, suggesting that gastric neurocircuitry involving the NTS was selectively impaired. Previous experimental studies suggest that CCK acts both peripherally and directly upon brainstem vagal circuits (Raybould and Tache, 1988; Fraser and Davison, 1992; Li and Rowland, 1995; Sayegh and Ritter, 2000; Baptista et al., 2005a,b, 2006; Holmes et al., 2009b). However, T3-SCI rats did not demonstrate a gastric efferent vagal response to central microinjection of CCK-8s into the DVC and the reduced sensitivity to centrally administered CCK-8s in the DVC persisted at 3 weeks after injury. Furthermore, whole-cell patch clamp recordings of NTS neurons from T3-SCI rats suggested a reduced activity of CCK-8s on synaptic inputs onto NTS neurons. Our preliminary data with peripheral and central administration of the prokinetic gut hormone, ghrelin, also reveals a reduced sensitivity within the NTS of SCI rats (Holmes et al., 2009a; Browning et al., 2010).

In summary, the reduction in vagal afferent responsiveness to mechanical and chemical stimuli, as well as the reduction in presynaptic glutamatergic inputs onto NTS neurons, suggests a generalized hyposensitivity of vagal afferent neurotransmission to the brainstem following SCI. Evidence of vagal afferent hyposensitivity has been identified in other GI pathobiological states (Hatanaka et al., 1997; Xue et al., 2009). In particular, Xue and colleagues suggest that part of the diminished visceral afferent sensitivity in an inflammation-induced model of functional dysmotility is mediated through an inducible NOS (iNOS) mechanism (Xue et al., 2009). However, this observation was limited only to afferents within the mesenteric arcade, and did not include the vagus. While levels of neuronal NOS are chronically diminished following experimental T9-SCI (Kabatas et al., 2008), preliminary data in T3-SCI rats suggests that GI iNOS levels are elevated in the days after SCI (Holmes, unpublished observation).

## Is diminished vagal sensory processing one culprit?

Whether these preliminary observations reflect a mechanism of SCI-mediated GI dysfunction requires further testing. The fact that SCI affects a neural circuit as spatially removed from the site of injury as vago-vagal control of the stomach presents an intriguing paradox. Since the main neural circuitry controlling the stomach remains physically intact after human and experimental SCI, why is gastric function compromised so persistently? Our evidence points to a persistent inhibition of vagally-mediated gastric reflexes which do not appear to involve sympathetic input to the stomach. While the neural mechanisms responsible for this post-SCI gastroinhibition remain obscure, recent reports provide a potential explanation.

### Neuroplasticity in the brain-gut axis

The spontaneous firing property of DMV neurons, mentioned previously, implies that alterations in firing rate can produce profound changes in gastric vago-vagal reflexes. Activation of NMDA and non-NMDA receptors in response to high levels of glutamate release is essential for the rapid transmission of feeding-relevant stimuli (Berthoud et al., 2001; Hornby, 2001). However, the visceral organ or function-specific wiring of brainstem neurocircuitry is complex, and the available permutations in synaptic organization confers a substantial degree of functional plasticity within GABAergic and glutamatergic synapses (see Babic et al., 2011). Recently an elegant model for vago-vagal plasticity has been put forward (Browning and Travagli, 2010). Essentially, these authors propose that cAMP levels within nerve terminals of GABAergic NTS neurons modulate receptor trafficking to the neuronal membrane, and hence regulate the ability of neurotransmitters of this synapse to be modulated (Browning et al., 2006; Browning and Travagli, 2009). Furthermore, cAMP levels may be regulated by vagal afferent input via group II metabotropic glutamate receptors (mGluR) known to be expressed within the DVC (Hay et al., 1999). Unlike glutamate release in response to digestion-relevant stimuli, tonic low level release of glutamate from vagal afferents keeps cAMP expression low due to a higher affinity of mGluR for glutamate. This activation of mGluR by low-level glutamate release confers greater NTS release of GABA, thus inhibiting gastric function, in addition to rendering the GABAergic neuron resistant to modulation (Browning et al., 2006; Browning and Travagli, 2007).

The NTS and DMV neurons are not the only point in the gut-brain axis where significant neuroplasticity occurs. ENS plasticity has been described in response to luminal contents (Soret et al., 2010) and disease processes (Huizinga et al., 2009; Mawe et al., 2009). As mentioned previously, vagal afferent fibers and cell bodies are sensitive to feeding related peptides such as CCK) (Appleyard et al., 2005; Baptista et al., 2005b). These vagal afferent neurons also demonstrate considerable plasticity to trauma (Zhang et al., 1996) as well as physiological stimuli (Burdyga et al., 2004, 2006a, 2008; de Lartigue et al., 2007). These physiological stimuli are proposed to switch vagal afferents between “fed” and “fasted” states (see Dockray and Burdyga, 2011) and provide one mechanism ultimately elevating cAMP levels in GABAergic neurons (Browning and Travagli, 2009).

### Future directions

Upon revisiting the vagal neurocircuitry controlling gastric function (presented in Figure 1), it is evident that there are several points where this pathophysiological cascade may occur. At the level of the GI lumen, diminished synthesis and/or release of GI peptides may represent a signaling failure following SCI (Figure 3.1). In addition to a persistent (up to 6 week post-SCI) reduction of gastric reflex responses to mechanical and peptidergic signaling, our observations demonstrate that *in vivo* CCK release following a mixed nutrient meal is diminished following SCI while iNOS mRNA expression is elevated (Holmes—unpublished observations). NO^−^ serves as an important regulator of CCK release in STC-1 cells (Mangel et al., 1996) as well as an endogenous modulator of vagal afferent sensitivity (Page et al., 2009). This suggests that in addition to smooth muscle relaxation, iNOS-derived NO^−^ may be implicated in the diminished release of GI peptides following SCI while also initiating a pathophysiological reduction of vagal afferent signaling at the level of the receptive fields.

**Figure 3:**
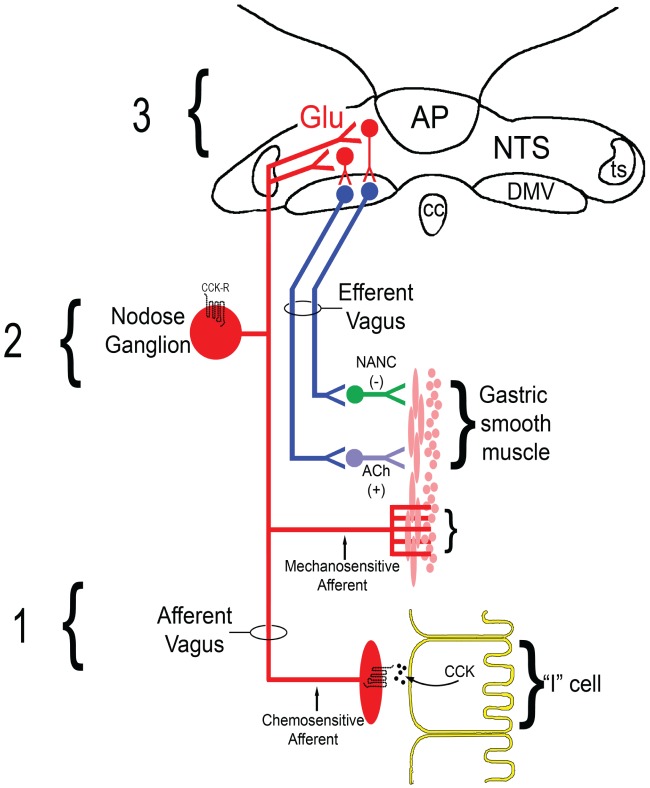
**Hypothesized schematic of diminished vagal sensory processing following T3-SCI.** (1) Impairments within GI enterocytes (for example CCK-secreting “I” cells) leading to a reduction in the synthesis or release of feeding-related GI peptides such as CCK may reflect a failure in transduction mechanisms at the level of the primary afferent. (2) Downregulation of appropriate receptors, which most likely occurs at multiple levels of vagal afferent neurons residing within the nodose ganglion [reviewed in Dockray and Burdyga (2011)], to feeding-related GI peptides diminishes glutamatergic signaling in the NTS-DMV leading to failed signaling at first synapse. Additionally, derangements in the transmission of gastric distension signals by mechanosensitive vagal afferents may also promote a parallel reduction in visceral stimuli which is independent of GI peptide release. (3) Ultimately, reduced signaling to NTS neurons permits unmodulated GABAergic inhibition of DMV motor outflow to the stomach that represents a failure of reflex integration at the second synapse in the vago–vagal circuit. Deficits at any one, or across all, of these levels will lead to gastric dysmotility and may limit the efficacy of potential therapeutic mechanisms.

Diminished CCK release entails additional pathobiological sequelae beyond failure to promote nutrient-mediated vagal afferent signaling. For example, gut permeability is a hallmark of numerous disease states and spinal transection has been shown to induce bacterial translocation within the gut (Liu et al., 2004). In a hemorrhagic shock model of inflammation and bacterial translocation, CCK-1 receptor activation of the vagus maintained intestinal integrity (Lubbers et al., 2009, 2010), presumably through activation of an α7-nicotinic receptor (α7-nAChR) anti-inflammatory mechanism (Luyer et al., 2005).

Independent of whether normal GI release of either CCK or ghrelin occurs after SCI, our observation that exogenous CCK fails to increase NTS *c-Fos* expression would indicate that the mechanism of gastric dysmotility may occur, at least in part, through reduced receptor expression and/or reduced excitability of vagal afferents. Neurophysiological changes in gastric vagal afferent fibers may form the second level of vagal afferent dysfunction in the development of post-SCI dysmotility (Figure 3.2). For example, vagal afferent reorganization occurs in response to surgically-induced gastric trauma (Phillips and Powley, 2005) and extends to alterations in the spontaneous firing rate of the afferent vagus (Miranda et al., 2009). The role of capsaicin-sensitive C-type fibers has long been recognized in GI reflex function, particularly CCK-mediated reflexes (Raybould and Tache, 1988; Sivarao et al., 1998). Additionally, vagal afferent neurons, which reside within the nodose ganglion, express CCK-1 receptors [aka. CCK type “A” receptors (Zhao et al., 2010)]. Identifying changes in vagal afferent responsiveness would provide a logical mechanistic explanation for our reported observation that exogenous peripheral administration of CCK induces diminished *c-Fos* expression following SCI (Tong et al., 2011). Similar observations have been provided for the action of ghrelin (Page et al., 2007) and expression of ghrelin receptors (Burdyga et al., 2006b; Page et al., 2007) and would extend to our observations after T3-SCI (Holmes et al., 2009a; Browning et al., 2010). These GI peptide-mediated changes are not the only mechanism by which visceral sensations fail to reach the brainstem as our data illustrates an impairment of mechanosensitive neurotransmission which also occurs following SCI.

Based upon the available data, our present research is further aimed at addressing the hypothesis that post-SCI reduction in vagal and/or NTS neuronal sensitivity to visceral signals biases GI brainstem circuits toward a tonic GABAergic inhibition of DMV efferent outflow to the stomach (Figure 3.3). Whether the mechanism of action is an inability of GI peptides to elevate cAMP levels in GABAergic neurons and/or nerve terminals after SCI or limited glutamatergic input that does little more than drive mGluR-mediated dampening of cAMP levels, it is the inability to modulate the inhibitory effects of this NTS-DMV GABAergic synapse that is most likely responsible for triggering gastric dysmotility following SCI.

### Translational perspective

Despite considerable evidence of upper GI dysmotility in the SCI population, the mechanisms remain poorly understood and require further study in order to develop evidence-based therapeutic strategies individuals with SCI. The conclusions of sympathetic involvement in the clinical report by (Fealey et al., 1984) still merits further investigation. However, there is growing evidence that derangements in gastric vagal neurocircuitry contribute to functional GI motility disorders in neutrally intact patients (Holtmann et al., 1998; Lunding et al., 2008; Manabe et al., 2011). Chronic changes in gut hormone levels, as well as the long term changes in vago–vagal gastric reflexes signaling are two universal targets for research in both intact and neurotrauma patients.

Ultimately, the failure within the brain-gut axis to respond to GI signaling pathways presents a clinical dilemma. Promising therapeutic strategies for other functional motility disorders may not necessarily translate to the SCI population or even across different regions of the GI tract (Holmes et al., 2009a; Browning et al., 2010; Ferens et al., 2011). Furthermore, while enteral feeding is associated with improved outcome in the critical care patient, conditions of enteral intolerance exist which may lead to increased mortality. Increasing gastric motility by pharmacological agents such as ghrelin mimetics offers considerable therapeutic potential. However, failure to understand the mechanisms which result in feeding intolerance may render therapeutic interventions ineffective if not detrimental. The unique sequelae of secondary injuries and pathologies spatially remote from the injury site, which are not limited to GI dysfunction, underscores the knowledge gaps which remain in our understanding of SCI.

### Conflict of interest statement

The author declares that the research was conducted in the absence of any commercial or financial relationships that could be construed as a potential conflict of interest.
